# Valuing health-related quality of life: systematic variation in health perception

**DOI:** 10.1186/s12955-018-0986-8

**Published:** 2018-08-02

**Authors:** Manuel Huber, Martin Vogelmann, Reiner Leidl

**Affiliations:** 1 German Research Center for Environmental Health, Institute for Health Economics and Health Care Management, Helmholtz Zentrum München, Postfach 1129, 85758 Neuherberg, Germany; 2 Wort & Bild Verlag Konradshöhe GmbH & Co. KG, 82065 Baierbrunn, Germany; 3 Munich Center of Health Sciences, Ludwig-Maximilians-University, Ludwigstr. 28 RG, 80539 Munich, Germany

**Keywords:** Systemic variation, Health-related quality of life, EQ-5D-5L, Utilities, Patient outcomes, Valuation, Value sets

## Abstract

**Background:**

Population-based value sets are widely used to transform health states into utilities, but may deviate from actual patient experience. Whether this occurs in a systematic way can be analyzed, in a first step, for respondents who do not report problems on the five domains of the EQ-5D-5L instrument in population studies.

**Methods:**

EQ-5D-5L results from three annual cross-sectional surveys (2012, 2013, and 2014) were filtered for participants who reported being problem-free. Continuous visual analog scale (VAS) scores, ranging from 0 (worst imaginable health) to 100 (best imaginable health) were then used to measure their actual health perception and to compare results with the proposed EQ-5D-5L value. A multiple linear regression model was used to identify possible risk factors for low VAS scores.

**Results:**

Some 3739 (61.5%) participants reported being problem-free. Their mean age was 41.1 years and mean VAS score was 91.9. Age and BMI were significantly associated with lower VAS scores. Age groups from 50 years onwards reported VAS means of 90.0 and below. Female gender and low education also had small but significant negative effects on patient experience. The presence of BMI class III as well as diabetes had the greatest negative effect on VAS results (− 9.0 and − 8.4) and reached the range of minimally important differences. Heart disease (− 6.2) and musculoskeletal disease (− 3.4) also had strong negative effects. The 25th percentile of VAS scores in our sample was 90.0, and the 50th percentile was 95.0.

**Conclusions:**

For some groups in population studies, especially older people with high BMI and those affected by specific diseases, no problems on all five domains of the EQ-5D-5L fails to reflect the respondents’ health perception as measured by the VAS.

## Background

In addition to mortality and clinical outcomes, health-related quality of life (HRQoL) can be used to evaluate and compare the effectiveness of healthcare interventions. HRQoL is measured by different instruments including the widely used EQ-5D [1–6]. The EQ-5D is a generic instrument consisting of five questions, referred to as dimensions. They are directed at different aspects of health (mobility, self-care, usual activities, pain/discomfort, and anxiety/depression). For each dimension, either three (3L version) or five (5L version) predefined answers exist. Answer possibilities range from no problem (=1) to extreme problems (=3 or 5, depending on the version) and make up the so called EQ-5D health state, a HRQoL profile (for reasons of compliance and standardization the term health state always refers to EQ-5D health state in this study). The visual analog scale (VAS) is also part of the EQ-5D-5L and is a continuous scale ranging from 0 (the worst health the responder can imagine) to 100 (the best health the responder can imagine). The VAS lets participants rate their overall health. Both the descriptive system and the VAS ask for health perception on the day of the survey. Self-reported EQ-5D data are often transformed into utilities to calculate quality-adjusted life years (QALYs) for cost-effectiveness analyses. The utilities are located on a scale anchored at 0 (dead) and ending at 1 (reporting no problems). The transformation into utilities is mostly achieved by applying country-specific value sets that assign fixed utilities for each health state [7]. These value sets are mostly created by asking representative samples of the population to value a number of hypothetical health states. Evidence suggests that population-based value sets may suffer from uninformed preferences of the general public [8]. Ratings are mainly done by applying the time-trade-off (TTO) method, which asks participants to trade time (e.g., years somebody is willing to die earlier) for perfect health. The most widely used value set for Germany and the EQ-5D-3L assigns a value of 1.00 to all respondents who report no problems (= 11111) in any of the five dimensions of the EQ-5D [9]. There are also value sets that are based on the health state currently experienced by the respondent. Respective value sets have been derived for the German population based on VAS valuations and the EQ-5D-3L [10] as well as on the EQ-5D-5L [11]. For the problem-free health state, these studies estimated a VAS value of 89.34 and 91.96 respectively. This lower valuation may reflect average health perception and may be used to calculate quality-adjusted survival, although it does not fulfill the requirements of the QALY concept [11]. Neither type of value set is able to account for valuation differences rooted in responder heterogeneity. Different patients often value the same health state differently. In case of systematic variation, a variety of utilities might thus be needed for the same health state.

In a first step, effects of patient heterogeneity on health perception can be analyzed for the problem-free health state that is reported most frequently in general population studies. For example, a comparison of EQ-5D-3L population studies in Germany, Spain, the UK, and the US found the share of this state to vary between 47 and 67% [12]. Even when using the more differentiated EQ-5D-5L, this health state represented a share of 61% in the general German population [13]. Despite selecting not having any problems, many older participants report significantly lower VAS scores than younger people who are problem-free [13]. While the age-based decline in health state and VAS score has been observed elsewhere [13–15], the decline in VAS scores among people reporting being problem-free should be investigated, as it reflects discrepancy between values assigned from a value set and an individual’s health perception (VAS score). Problems with the EQ-5D deviating from the best health state are known [16]. Taking the example of Germany, this study investigates the relationship between the problem-free health state of the EQ-5D-5L and self-reported VAS scores. Sociodemographic parameters and other parameters such as disease affliction are evaluated to assess their influence on variation in health perception.

## Methods

### Study population

Three cross-sectional surveys (2012, 2013, 2014) were used in this study. These surveys were conducted annually. To ensure that participants are representative of the general German population, a random route procedure was used to select around 2000 participants per year. The random route procedure and additional survey content are described in more detail elsewhere [13].

### Survey content

The yearly surveys include questions about healthcare habits as well as healthcare status of participants and have incorporated the EQ-5D-5L since 2012. The survey includes questions regarding sociodemographic data, disease affliction, healthcare utilization, and HRQoL. Evaluated parameters, among others, include age, sex, height, weight, education (low education: primary school; medium education: secondary school certificate; high education: general qualification for university entrance or advanced technical college entrance qualification), job status (self-employed, public servant, employee, worker, unemployed), and the presence of disease. Disease affliction and other parameters are based on self-report by participants and were not assessed by clinical means. Participants were asked to name diseases they had suffered from during the last 3 months before the survey. Cold, flu, migraine and tooth problems were considered as acute occurrence, the other diseases were considered as chronic.

### HRQoL

The EQ-5D-5L is a generic tool that can be used among different fields of indication. It is well accepted by healthcare authorities such as the National Institute for Health and Care Excellence (NICE) and was used in each of the three surveys. For the purpose of this study, the health state 11111 (no problem in any dimension) is referred to as being problem-free. In addition to the five dimensions, the respondents were also asked to rate their current health on the VAS.

### Data analysis

Datasets from 2012, 2013, and 2014 were merged and tidied. Participants with N/As for the EQ-5D-5L descriptive system and/or VAS result were removed. Body mass index (BMI) was grouped based on WHO classification recommendations [17]. Only diseases that were stated by more than five survey takers per gender were included in the analysis to improve the clarity of the paper as well as the clinical transferability of the results irrespective of gender. While linear models assume homoscedasticity of error terms, this assumption is often violated by HRQoL data, as a majority of participants often report very good health states. The Breusch–Pagan test was highly significant for our linear model, indicating that variance of regression errors is heteroscedastic. To receive heteroscedasticity-consistent standard errors, we calculated sandwich estimators [18]. Robust regression is based on less restrictive assumptions and represents an alternative to least squares regression when error terms are heteroscedastic [19]. All analyses are based on the software environment R [20], version 3.4.0. Figures were created using the package ggplot2 [21].

## Results

The total sample included 6074 observations, of which 3739 (61.5%) participants reported to be problem-free (11111). Table 1 shows the sample characteristics. The mean age was 41.1 years and the mean VAS score was around 91.9. The mean VAS score for problem-free participants who reported not having any disease (not shown here) is 93.51 (*n* = 2493). Less than a quarter had high education (general qualification for university entrance or advanced technical college entrance qualification). The majority were living together with other household members. Females suffered from more chronic diseases. More than three-quarters of participants were either employees or workers. Overall, 12.2% did not answer the question about employment. Of those participants who stated their height and body weight, 56.8% had a normal BMI and 41.1% were considered to be overweight/obese. There were 12 participants with diseases of the eye, six participants with diseases of the ear, and only four participants with depression (not shown here). The most common chronic diseases were musculoskeletal diseases and hypertension. Variation of observed variables among the three survey years was generally low [13, 22].

**Table 1:** Sample characteristics

Characteristic	Male	Female	Total
Population	1911 (51)	1828 (49)	3739 (100)
Mean age (years)	41.3 (±15.5)	40.8 (±15.4)	41.1 (±15.5)
Mean VAS	92.4 (±9.0)	91.3 (±9.4)	91.9 (±9.2)
Education			
Low	611 (17.3)	533 (15.1)	1144 (32.4)
Medium	712 (20.2)	844 (24.0)	1556 (44.2)
High	470 (13.3)	354 (10.0)	824 (23.3)
Living with partner			
No	874 (23.4)	779 (20.8)	1653 (44.2)
Yes	1037 (27.7)	1049 (28.1)	2086 (55.8)
Household size			
> 1 person	1316 (35.2)	1405 (37.6)	2721 (72.8)
1 person	595 (15.9)	423 (11.3)	1018 (27.2)
Employment			
Self-employed	193 (5.2)	97 (2.6)	290 (7.8)
Public servant	79 (2.1)	41 (1.1)	120 (3.2)
Employee	778 (20.8)	1277 (34.1)	2055 (54.9)
Worker	622 (16.6)	178 (4.8)	800 (21.4)
Unemployed	4 (0.1)	13 (0.3)	17 (0.4)
Not available	235 (6.3)	222 (5.9)	457 (12.2)
BMI			
Mean BMI (SD)	25.23 (±2.9)	23.79 (±3.9)	24.54 (±3.6)
Underweight (< 18.5)	5 (0.1)	68 (2.0)	73 (2.2)
Normal weight (18.5–24.9)	891 (26.5)	1018 (30.3)	1909 (56.8)
Overweight (25–29.9)	786 (23.4)	406 (12.1)	1192 (35.5)
Obesity class I (30–34.9)	73 (2.2)	80 (2.4)	153 (4.6)
Obesity class II (35–39.9)	12 (3.6)	13 (0.4)	25 (0.7)
Obesity class III (≥40)	3 (0.1)	6 (0.2)	9 (0.3)
Disease			
Allergy	10 (0.3)	30 (0.8)	40 (1.1)
Asthma	8 (0.2)	15 (0.4)	23 (0.6)
Bladder	2 (0.1)	23 (0.6)	25 (0.7)
Cold^a^	135 (3.6)	176 (4.7)	311 (8.3)
Diabetes	19 (0.5)	25 (0.7)	44 (1.2)
Flu^a^	48 (1.3)	50 (1.3)	98 (2.6)
Gut	46 (1.2)	56 (1.5)	102 (2.7)
Heart	12 (0.3)	11 (0.3)	23 (0.6)
Hypertension	47 (1.3)	69 (1.8)	116 (3.1)
Migraine^a^	79 (2.1)	157 (4.2)	236 (6.3)
Musculoskeletal	73 (1.9)	63 (1.7)	136 (3.6)
Skin	12 (0.3)	19 (0.5)	31 (0.8)
Tooth^a^	28 (0.7)	35 (0.9)	63 (1.6)
Thyroid	5 (0.1)	38 (1.0)	43 (1.1)

Percentage or SD in brackets; *BMI* body mass index; low education: primary school; medium education: secondary school certificate; high education: general qualification for university entrance or advanced technical college entrance qualification

^a^considered as acute diseases, other diseases considered as chronic

**Fig. 1:**
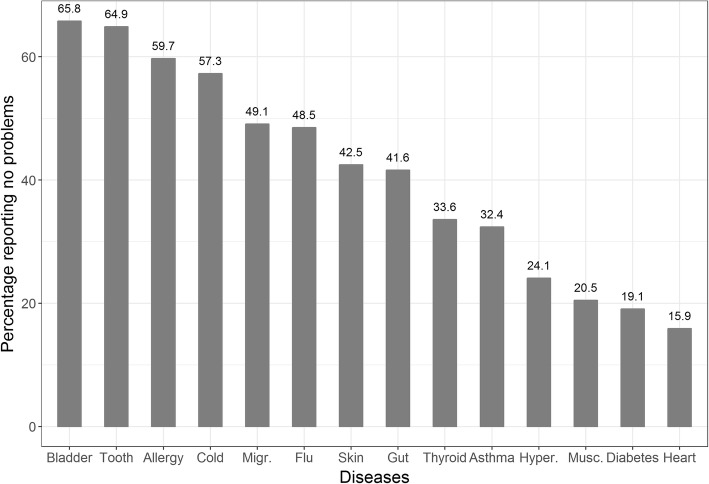
Percentage of participants with specific diseases reporting no problems, bar chart. Note: Percentage of people who are affected by diseases but report no problems, e.g., 59.7% of survey participants who reported having an allergy also reported having no problems in the five dimensions of the EQ-5D-5L (= 11111). Hyper.: Hypertension; Migr.: Migraine; Musc.: Musculoskeletal disease

The share of participants who are affected by specific diseases and report no problem in the descriptive system differs greatly among diseases (Fig. 1). While around 60% with bladder disease, dental disease, or allergy report being problem-free, only around 20% do so when they are affected by diabetes, musculoskeletal disease, or heart disease.

**Fig. 2:**
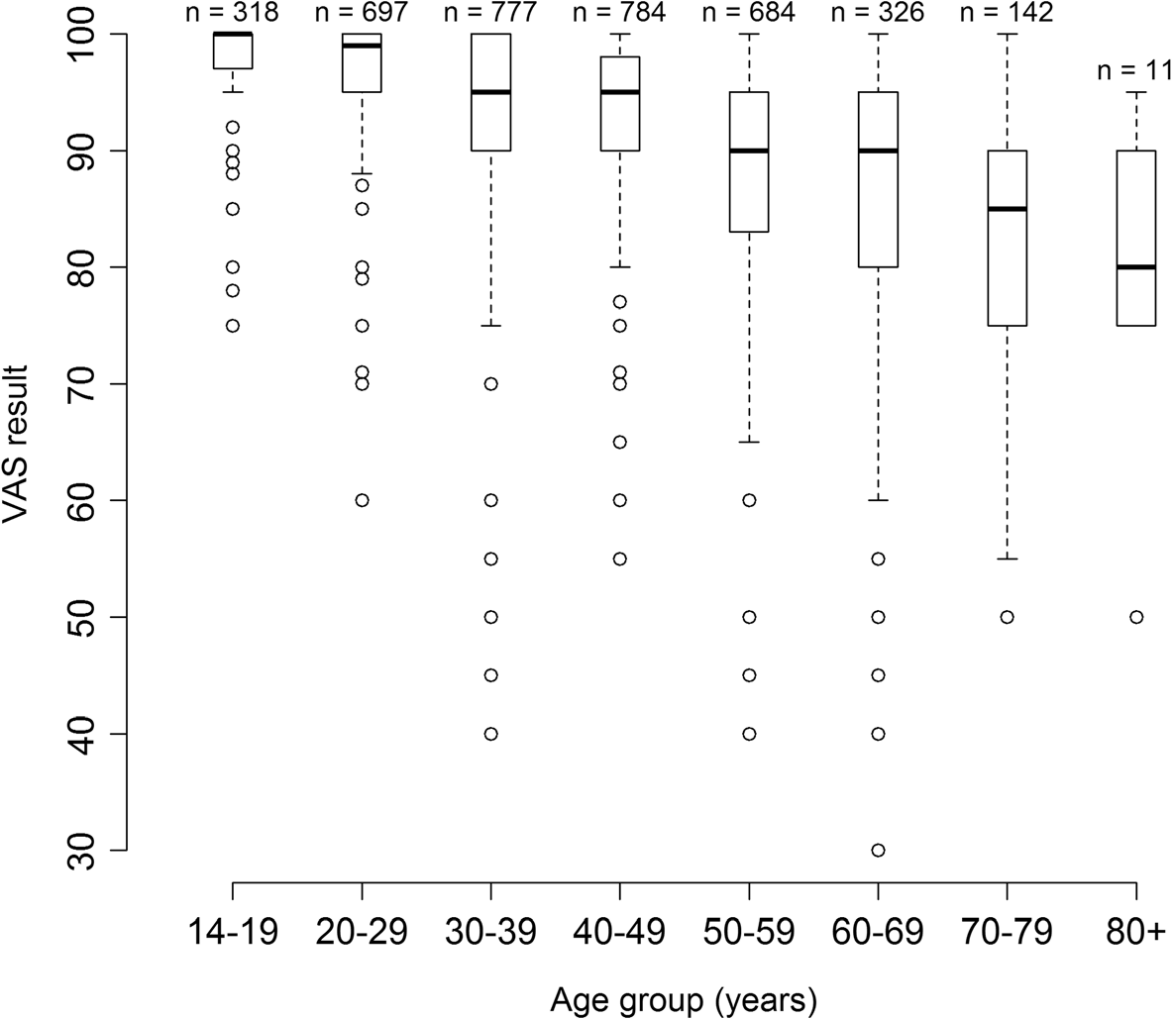
Boxplots of VAS results stratified by age group

Overall, median VAS scores decline and interquartile ranges increase by age group (Fig. 2). Median VAS scores are quite similar for age groups 14–19 and 20–29 years (around 98), 30–39 and 40–49 years (around 95), as well as 50–59 and 60–69 years (around 90). Outliers are present in every age group. The sample size of age group 80+ years is small.

Table 2 shows the results of the multiple linear regression model with respective sandwich estimators. Female gender and older age are significantly associated with lower VAS scores. Overweight and obesity classes I + III are associated with lower VAS scores, while being underweight has no significant influence. Employment status is borderline but currently not significant. Medium and high education are associated with higher VAS scores. Obesity class III and diabetes have the strongest negative significant influence and lower VAS scores by 9.0 and 8.4 points respectively.

**Table 2:** Parameter estimates, robust regression, VAS as dependent variable

	Estimate	Std. error	Pr(>|t|)	Significance
(Intercept)	98.3150	2.2046	0.0000	***
Sex	−0.8091	0.2861	0.0047	**
Age	−0.2063	0.0115	0.0000	***
Education				
Low	Reference			
Medium	0.7643	0.3260	0.0191	*
High	0.9874	0.3767	0.0088	**
Employment status				
Unemployed	Reference			
Employed	4.0233	2.1912	0.0664	.
No answer	3.8399	2.1863	0.0791	.
Public servant	4.1347	2.3132	0.0740	.
Self-employed	3.9145	2.2546	0.0826	.
Worker	3.9608	2.2051	0.0725	.
BMI				
Normal weight	Reference			
Underweight	0.7083	0.6040	0.2410	
Overweight	−1.6191	0.3009	0.0000	***
Obesity class I	−3.1870	0.8692	0.0002	***
Obesity class II	−3.1904	1.6789	0.0575	.
Obesity class III	−9.0364	3.0929	0.0035	**
Disease affliction	Binary			
Allergy	0.1723	1.9057	0.9280	
Asthma	−3.6867	2.9752	0.2154	
Cold	1.0383	1.2530	0.4073	
Diabetes	−8.4187	2.2352	0.0002	***
Flu	1.4430	1.3633	0.2899	
Heart disease	−6.1609	2.8770	0.0323	*
Hypertension	−2.6707	1.5183	0.0787	.
Gut	0.9271	1.5420	0.5477	
Migraine	−0.4713	1.2814	0.7131	
Musculoskeletal disease	−3.3929	1.5141	0.0251	*
Periodontal disease	0.5671	1.4143	0.6885	
Skin disease	−1.2974	2.5502	0.6110	
Thyroid disease	−1.9188	2.4029	0.4246	
One disease	−3.2772	1.2155	0.0070	**
Two diseases	−3.8294	2.1846	0.0797	.
Three diseases	−4.8167	3.5382	0.1735	
Four diseases	−8.2427	5.5741	0.1393	

Significance levels:. *p* < 0.1, * *p* < 0.05, ** *p* < 0.01, *** *p* < 0.001

**Fig. 3:**
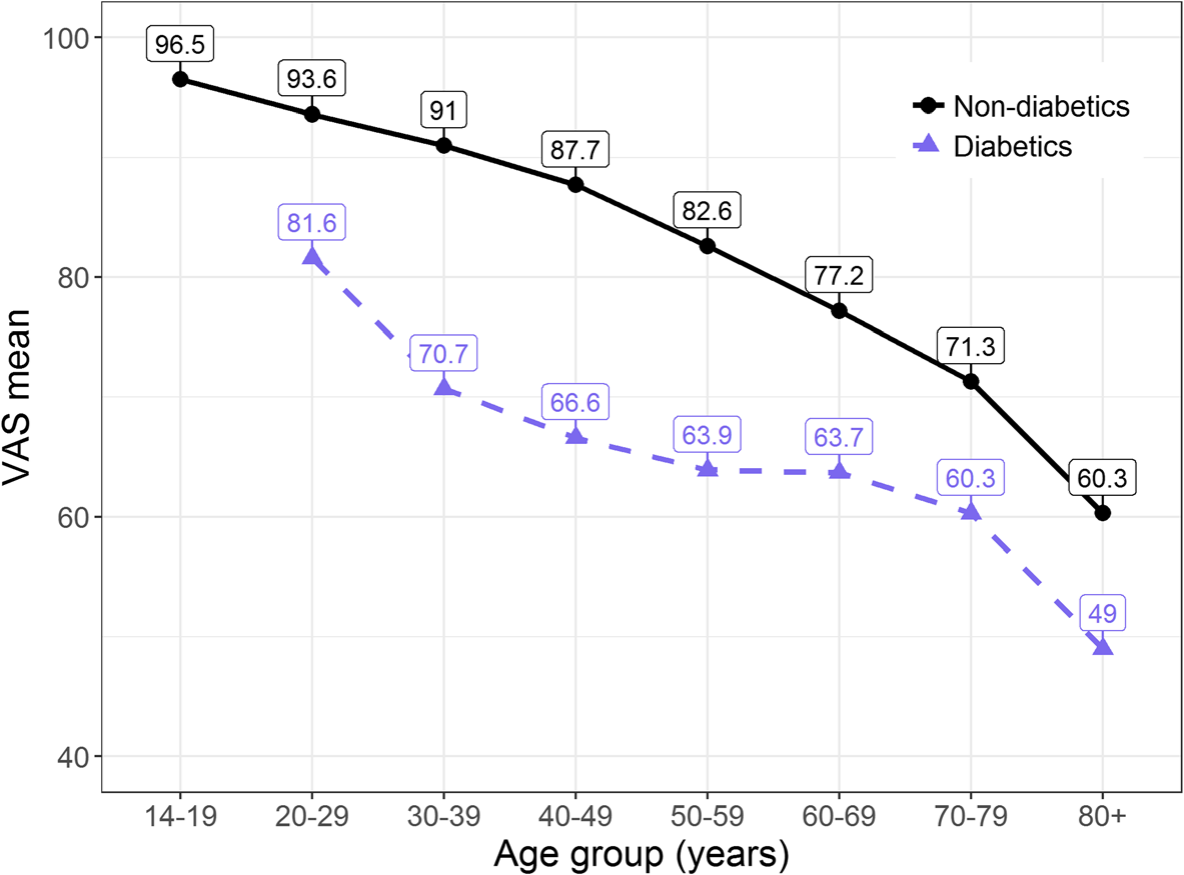
VAS mean by age groups, non-diabetics vs. diabetics, problem-free only. Note: There were no diabetics in age group 14–19 years

Diabetes has the strongest significant negative effect on VAS results. Figure 3 illustrates VAS means of diabetics and non-diabetics, who reported being problem-free, stratified by age group. While there are no diabetics in age group 14–19 years, the difference in VAS means increases from around 10 points in age group 20–29 years to around 20 points for age group 30–59 years, before it narrows again.

## Discussion

We clearly show that VAS scores among people who report to be problem-free suffer from systematic variation. While a majority of young people report VAS scores of 100, mainly older people and participants with certain diseases do not. Older adults with low education, obesity, and chronic diseases such as diabetes or cardiac disease seem to be especially prone to reporting low VAS scores despite stating no problems in the EQ-5D-5L descriptive system.

### Additional factors influencing VAS responders

One possible explanation for this discrepancy is the weakness of the five dimensions in reflecting all limitations of associated diseases. Respective evidence is available for macular degeneration, hearing disorders, and psychotic disease [23–28]. However, the number of people with self-reported visual or hearing disorders or depression was very low in our sample. One basic shortcoming of the EQ-5D-3L compared with the 15D instrument is its problem in deviating from 11111, especially for alcohol use disorders, migraine, psoriasis, and disturbing allergy [16]. Because we use the 5L version and have differing disease focus, the impact of this problem on our observations can hardly be assessed. 179 respondents out of a sample of 436 participants of the UK general population stated aspects that were important but missing from the five dimensions of the EQ-5D [28]. Sensory deprivation (50 responses) and mental health (72 responses) were mentioned the most. Overall, simple having or not having a disease, irrespective of symptoms, may represent an important aspect of health that explains lower VAS results in some people and should therefore be taken into consideration. 15 respondents also complained that a reference for communication was missing, 13 missed a dimension for lifestyle and fitness, 10 missed non-health outcomes (work, financial stability), 15 missed a dimension regarding relationships, loneliness and sociability, 6 missed spirituality and 4 tiredness. These aspects may influence VAS responses but based on current data of this study it is not possible to decipher their degree of influence. Disease-specific sensitivity problems of the EQ-5D-3L and 5L are currently addressed by implementing so-called bolt-on items. They are currently available for psoriasis, disorders of the eye and ear, or sleeping disorders [25, 29–32]. A bolt-on item for respiratory disease is currently under development [33]. Bolt-on items take the same form as other EQ-5D questions but focus on additional disease dimensions, where the performance of the EQ-5D is currently suboptimal. The introduction of the EQ-5D-5L version may have improved performance to some degree, on account of increased sensitivity and fewer ceiling effects, especially in older people [34, 35]. Nevertheless, the five EQ-5D dimensions remain the same, and health problems not reflected in these dimensions are likely missed in the new version as well. Accordingly, the 50th percentile of VAS scores in our sample of problem-free participants is 90 for age group 50–69 years and 85 for age group 70+ years.

Shah et al. [36] also evaluated the influence of wording used for being problem-free, on valuation outcomes in TTO studies. Over 40% of Shah et al.’s respondents stated that important aspects of health were not covered by the five EQ-5D dimensions. Moreover, the authors stated: “224 respondents (50.6%) self-reported as being in health state 11111. Of these 224 respondents, 187 (83.5%) self-reported an EQ-VAS score of less than 100 (…) The mean (median) EQ-VAS score for respondents self-reporting as being in 11111 was 89.1 (90).” This confirms our findings; the VAS is an important supplement to the descriptive system and should not be disregarded as it contains information that is not reflected by currently assigned values, which are only based upon the descriptive part of the EQ-5D, especially the value for 11111.

### Current value sets for the EQ-5D-5L

An overview of available official value sets for the EQ-5D-5L and their respective values for the problem-free health state may help to assess the extent of this issue. Eight value sets for eight different countries are currently (August 2017) listed on the EuroQol site [7]. At least six of these value sets (China, England, Indonesia, Korea, Netherlands, Uruguay) propose 1 as the value for 11111 [37–42]. Interestingly, Luo et al. [41] point toward another issue with some value sets: “Although most previous studies of this kind chose to adjust only the value for 11111 (to 1), we elected to adjust all the values to preserve the relative utility of all the health states.” Therefore, to fulfill the criteria of the QALY concept, some authors manually set the value for 11111 to 1, while their actual models proposed lower utilities for the best health state. Remaining values were not adjusted in most of these studies, and it is unclear which approach should be preferred. Xie et al. [43], in their value set for Canada, extrapolated their preferred model and calculated 0.949 to be the value for being problem-free. They state that optimal health may go beyond not having any problems in the five dimensions of the EQ-5D-5L. Our findings support this conclusion and point out that even more differentiation might be necessary. Before choosing a value set, modelers and decision makers should be aware of their perspective and respective implications. From a population perspective, assigning utilities of 1 to all people in the problem-free health state may be correct but from a patient perspective it frequently is not.

### Impact

The focus on patient outcomes is increasing in Germany [44] and around the world [45]. For example, the National Health Service requires providers to collect patient-reported outcome measures (PROMs) for several elective procedures and, overall, PROMs are becoming more and more important for healthcare providers and decision makers [46]. However, the observed systematic variations in individual health perception are not captured by value sets based on health state descriptions as, for one health state, these will always attribute one value (both utility-based value sets and experience-based value sets). As shown, systematic deviations in valuations can reach ranges of clinically minimally important differences [47, 48] and thus affect assessment in a substantial way. Furthermore, quality of life loss was found to vary by age for respondents reporting diabetes, reaching especially high levels in those between 30 and 60 years of age. If the systematic deviations in valuations are considered relevant both in size and with regard to the research question, it is necessary to test whether or not an EQ-5D value set is a valid measure in a target group of responders; this applies especially if older age, higher BMI, and specific diseases prevail. Disregarding this systematic variation in valuation may lead to overestimation as well as underestimation of the health gains that are achievable by intervention. Moreover, the current health status of patients may be misrepresented when VAS scores are disregarded.

### Strength and limitations

One strength of this study is the random route procedure that was used to select participants. This sophisticated method improves the representativeness of the study population and minimizes selection bias. Moreover, to our knowledge, this is the first study to evaluate systematic variation between the assigned value for 11111 and actually perceived health in Germany. One limitation of this study is that certain biases may have influenced results. For example, respondents are often more inclined to report non-extreme answers on rating scales (central tendency bias). This is especially important for the VAS, as it ranges from 0 to 100 and does not include descriptions for predefined sections of the scale. However, if the central tendency bias is present, it should exist throughout all age groups. This is not the case in our study, with young people mostly reporting VAS scores of 100 and older people tending to report lower scores. Unless the central tendency bias increases with older age, its presence cannot be confirmed in our data. Evidence exists, that increased cognitive loads increase the central tendency bias [49] but we are unaware of evidence evaluating cognitive loads based on age. Another limitation of this study is the lack of clear definitions for certain diseases, e.g., the term skin disease subsumed a wide variety of skin diseases that differ in severity and impact on patient HRQoL. Moreover, significant predictors that go beyond our evaluated independent variables were missed, and thus could not be used to explain the variation. Other aspects of well-being like aesthetics, cognitive capabilities, relationships or communication may influence VAS responses. While this issue certainly deserves more research, this was not possible within the scope and data of this study. Finally, the variation has “only” been shown for the most frequent health state—which is highly relevant in general population surveys—but it remains unclear to what extent other health states are affected by the phenomenon. Furthermore, the question of why individual health perception differs between a descriptive system and VAS is not the focus of this paper. More research may be needed in this regard. Our research goal was to evaluate the existence of systematic variation among health perceptions and what the respective predictors look like in our sample.

## Conclusion

In an era in which individual health outcomes have become the new focus of healthcare, adequate valuation for health states including different population groups becomes necessary. Focusing on the large share of the general population not reporting any problem in the EQ-5D descriptive system, a single value for all has been found not to adequately reflect actual health valuation for several subgroups, especially for older people with high BMI and those affected by specific diseases. If such heterogeneity is present and impacts study results, individual health perception should be considered, and VAS results should not be disregarded.

## Abbreviations

HRQoL: Health-related quality of life
PROMs: Patient-reported outcome measures
TTO: Time-trade-off
VAS: Visual analog scale
